# Combined effect of interstitial-substitutional elements on dislocation dynamics in nitrogen-added austenitic stainless steels

**DOI:** 10.1038/s41598-024-54852-w

**Published:** 2024-02-22

**Authors:** Yasuhito Kawahara, Shunya Kobatake, Kenji Kaneko, Taisuke Sasaki, Tadakatsu Ohkubo, Chikako Takushima, Jun-ichi Hamada

**Affiliations:** 1 Department of Materials, Kyushu University, 744 Motooka, Nishi, Fukuoka, 891-0395 Japan; 2 National Institute for Materials Science, 1-2-1 Sengen, Tsukuba, 305-0047 Japan; 3 Research & Development Center, Nippon Steel Stainless Steel Corporation, 3434 Shimata, Hikari, 743-8550 Japan

**Keywords:** Metals and alloys, Transmission electron microscopy

## Abstract

Combined addition of interstitial-substitutional elements has been acknowledged to contribute to the increase in the strengths of steels. For further improvements in mechanical properties, their atomic-scale interaction mechanisms with dislocations are required to be examined. In this study, both high-resolution transmission electron microscopy and atom-probe tomography were used to correlate interstitial-substitutional elements with dislocation characteristics in austenitic stainless steels. Three types of dislocation core structures are identified and associated with their strain fields as well as N and Cr atoms in the N-added steels. It is revealed that N atoms interact elastically with the dislocations, followed by the segregation of Cr atoms via the chemical interaction between N and Cr atoms. This insight significantly improves the understanding of the multiple alloying mechanism in metallic materials such as interstitial alloys and high-entropy alloys.

## Introduction

Alloying is one of the most fundamental and common ways to control the properties of metallic materials, such as strength^1–3^, ductility^4–6^, and corrosion resistance^7–9^. The multiple addition of alloying elements often dramatically enhances these properties^10–12^, and thus much attention has been paid to identify roles of alloying elements on the properties by developing atomic-scale characterization and computational materials science methodologies^13–15^. The main objective of these methodologies is to correlate the extended defects with the alloying elements, such as interstitials, substitutional, and clusters^13–15^, and to establish the interaction mechanisms between the defects and the alloying elements.

Austenitic stainless steels, Fe–Cr–Ni alloys, are the typical metallic materials with multiple alloying elements, which exhibit excellent strengths over a wide range of temperatures^16^. N addition enhances mechanical strength^17,18^, as well as the work-hardenability^19^ by changing the dislocation configuration from wavy to planar^19,20^. Hitherto, three mechanisms have been proposed to explain the origin of this planarization. One is that N atoms simply stabilize stacking-faults (SFs)^19,21^, where the dislocations are dissociated into two partial-dislocations, resulting in the suppression of the cross-slips. Another is the glide plane softening mechanism^20,22–24^, where leading dislocations destroy the pre-existing N–Cr chemical pairs^18^, followed by the formation of easy slip planes for subsequent dislocations. The other one is the interstitial-substitutional (I–S) effect proposed by Monma et al^25,26^., where N is first elastically segregated to the dislocations, followed by the segregation of Cr via the attractive chemical interaction with N.

Although the origin of the planar slips has been proposed as these three mechanisms, it is not clarified yet which mechanism contributes to the planar slips. To understand the superior work-hardenability of N-added austenitic stainless steels, it is necessary to reveal the origin of the planar slips, which should depend on the interaction mechanisms between N atoms and the dislocation characteristics. This study shows the correlations of dislocation characteristics with segregated impurities as well as their local strain fields in the austenitic stainless steels deformed at high temperature by both transmission electron microscopy (TEM) and atom-probe tomography (APT). We find that the I–S effect contributes to the dislocation dynamics during the deformation, where the N and Cr atoms stabilize three types of dislocations: edge-dislocations, partial-dislocations, and Lomer-Cottrell locked dislocations (LCDs), and result in superior work-hardenability via the planar slip of the dislocations.

## Results

### Mechanical properties and microstructural characterizations by TEM

**Figure 1:**
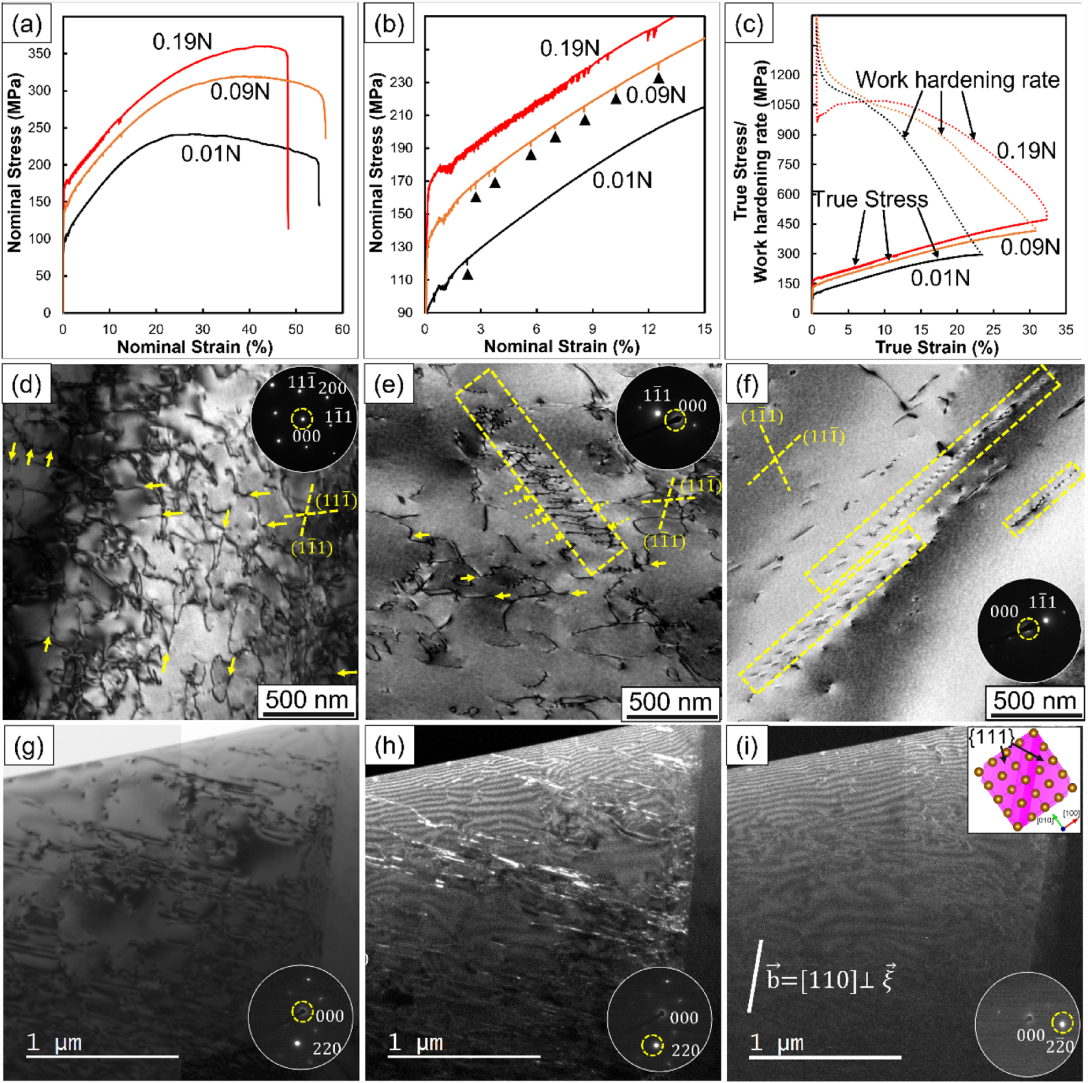
Tensile properties and deformation structures of the alloys. (**a**) Nominal stress–strain (S–S) curves, (**b**) the S–S curves up to 15% strain, and (**c**) changes in true stress and work-hardening rate as a function of the true strain in 0.01N, 0.09N and 0.19N. (**d**–**f**) BF-TEM images of the dislocation structures in 0.01N, 0.09N and 0.19N, respectively. (**g**–**i**) BF-TEM and a series of weak-beam DF-TEM observations of 0.19N.

Experiments were performed on high-temperature and high-corrosion resistant austenitic stainless steels, Fe–20Cr–14Ni–0.05C–3Si–0.8Mn–0.1Mo–0.2Cu–0.1V–0.02Al–0.003O–0.03P–0.0007S steels with 0.01, 0.09 and 0.19 of N in wt% or (Fe–20Cr–12Ni–0.21C–6.0Si–0.81Mn–0.05Mo–0.17Cu–0.11V–0.055P–0.001S steels with 0.039, 0.35 and 0.73 of N in at%), indexed as 0.01N, 0.09N and 0.19N, respectively. Figure 1a shows the nominal stress–strain curves at 973 K with a strain rate of 1.4 × 10^−3^ s^−1^, where the N addition raised both yield and tensile strengths. The yield strengths were 94.8 MPa, 136 MPa and 169 MPa for 0.01N, 0.09N and 0.19N, respectively, enhanced by the solid solution hardening of N^27^. A larger increase in tensile strength is also observed due to the enhancement of work-hardenability by N addition^19^. Figure 1b shows the nominal S–S curve up to the 15% strain range, where the serrated flow was frequently confirmed as N content increased, as indicated by the black arrows, indicating the occurrence of dynamic strain aging. As shown in Fig. 1c, N addition enhanced the work-hardening rate especially in the range between 7 and 30% strain. Up to 7% strain, the work-hardening rate of 0.19N was lower than that of the others, possibly due to the yield drop caused by dynamic strain aging.

Figure 1d, e and f show bright-field (BF)-TEM images of dislocations viewed along [011] of the matrix in 0.01N, 0.09N and 0.19N deformed up to 5% strain at 973 K, respectively. As indicated by yellow solid arrows in Fig. 1d, some segments of dislocations were located on $(11\overline{1 })$ and $(1\overline{1 }1)$, and others were on $(111)$ and $(\overline{1 }11)$, suggesting that cross-slips were frequently occurred in 0.01N. In the case of 0.09N, as indicated yellow solid arrows, cross-slips were recognized, as confirmed in 0.01N, and a weak fringe-contrast possibly due to SFs is seen in the edge portion of dislocation arrays, as indicated yellow dotted arrows in Fig. 1e. Planar slips were recognized in 0.09N, and frequently confirmed in 0.19N, as surrounded by the yellow dotted square in Fig. 1e, f, respectively. Figure 1 g shows a two-beam BF-TEM image, and Fig. 1h, i show weak-beam dark-field TEM images of 0.19N viewed along [001] of the matrix with different excitation conditions, $\overrightarrow{{\text{g}}}$=$[220]$ and $[2\overline{2 }0]$, respectively. As shown in Fig. 1g, h, linear dislocations were observed frequently on the slip planes. Figure 1i shows that these contrasts disappeared under the specific excitation condition, $[2\overline{2 }0]$, and $\overrightarrow{{\text{g}}}\cdot \overrightarrow{{\text{b}}}$ analysis suggested that these linear dislocations were edge-dislocations.

**Figure 2:**
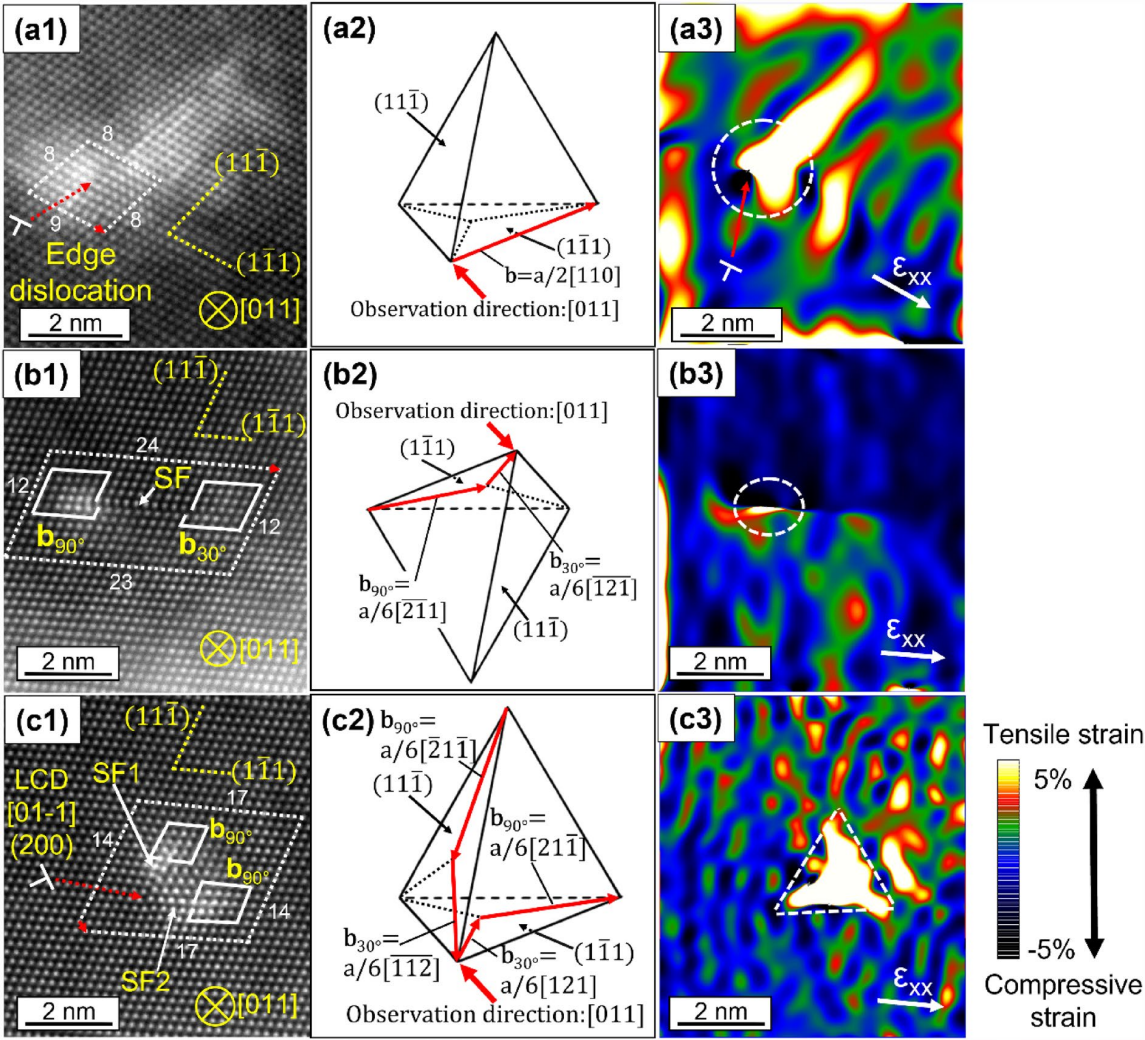
The structures and strain maps for dislocation cores in 0.19N. (**a1**-**c1**) LAADF-STEM images of edge-dislocation, SF and LCD, respectively. White dotted and solid lines are the Burgers circuits around the dislocation cores of extra-half planes and partial-dislocations, respectively, where red arrows exhibit the lattice imperfections, which correspond to Burgers vectors. The red dotted arrows indicate the core location. (**a2**–**c2**) Thompson’s tetrahedra used for determination of the Burgers vectors of edge-dislocation, partial-dislocation and LCD, respectively. (**a3**–**c3**) Strain maps by GPA analysis for edge-dislocation, partial-dislocation and LCD, respectively. The x directions are predefined as [$21\overline{1 }$] for all types of dislocations.

Figure 2 shows low-angle annular-dark field (LAADF)-STEM images, Thompson’s tetrahedra, and strain maps of three dislocation core structures in 0.19N. Consistent with the $\overrightarrow{{\text{g}}}\cdot \overrightarrow{{\text{b}}}$ analysis, edge-dislocation was determined from the presence of an extra-half plane^28^, as shown in Fig. 2a1. In addition, Fig. 2b1 shows the presence of the partial-dislocation on the edge of SF^28,29^, where the atomic shift can be easily recognizable, as indicated by the white solid arrow. The presence of an extra-half plane suggests that an edge-dislocation was dissociated to form partial-dislocations, where one partial was confirmed as bright-contrast while another wan not. In the projected plane viewed along [011] of the matrix, as shown in Fig. 2b2, one partial, ${\overrightarrow{{\text{b}}}}_{90^\circ }$, exhibits larger amount of atomic shift, compared with another partial, ${\overrightarrow{{\text{b}}}}_{30^\circ }$, corresponding to the bright contrast confirmed around partials in Fig. 2b1. As shown in Fig. 2c1, c2, the intersection of SFs on $(11\overline{1 })$ and $(1\overline{1 }1)$ planes led to the formation of V-like configurations^28,30^, forming a sessile stair-rod dislocation by the following reaction: ${\text{a}}/6[\overline{112 }]+{\text{a}}/6[121]\to {\text{a}}/6[01\overline{1 }]$, corresponding to an LCD^28,30^.

**Figure 3:**
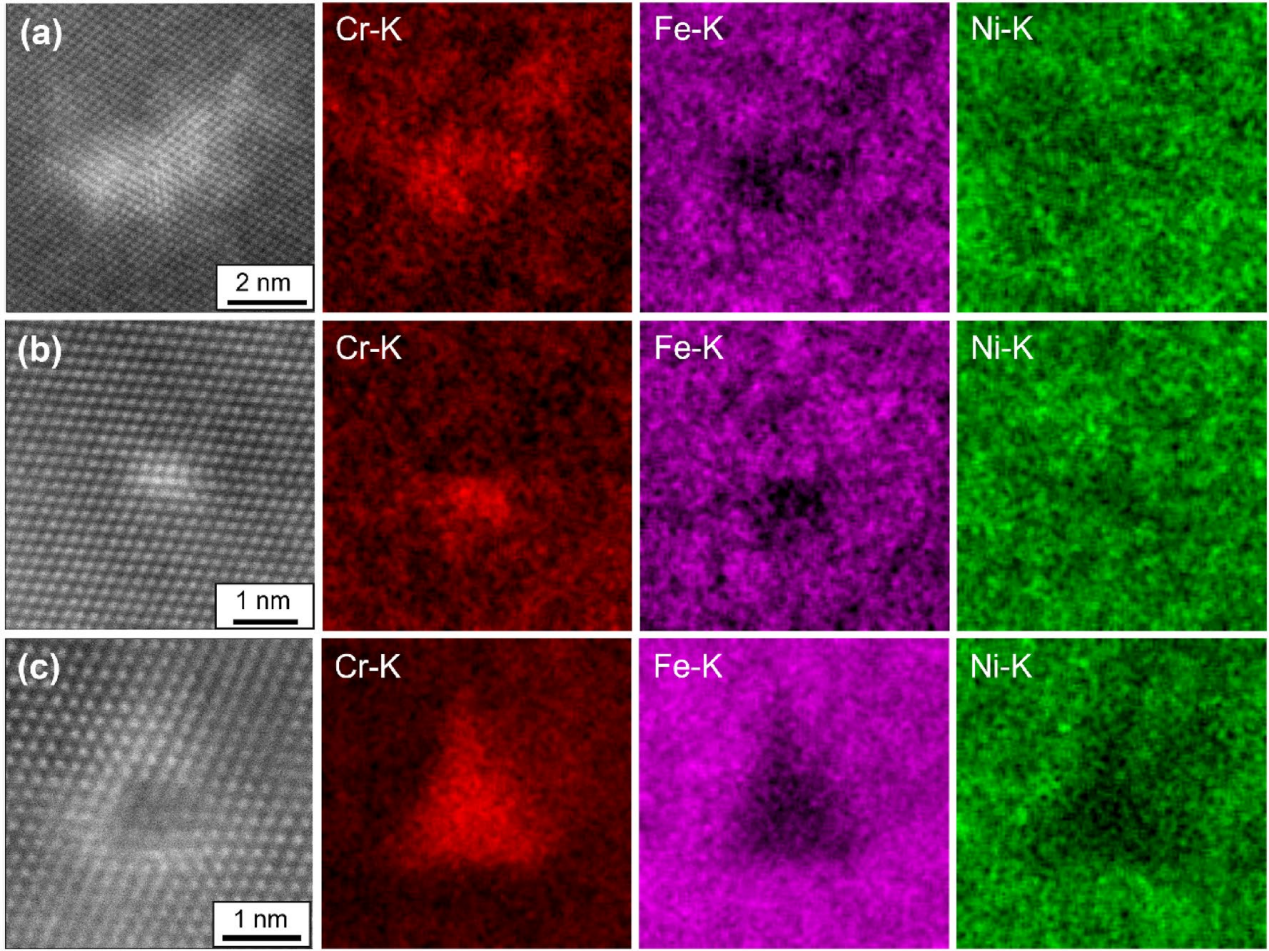
Magnified LAADF-STEM images and elemental maps of the Cr–K, Fe–K and Ni–K for edge-dislocation (**a**), partial-dislocation (**b**) and LCD (**c**). EDS maps were taken from regions where GPA analysis were carried out in Fig. 2.

Tensile strain fields were present around the dislocation cores in the case of the edge-dislocation and partial-dislocation, as shown in Fig. 2a3, b3 respectively, while LCD exhibited the triangular tensile strain field, as shown in Fig. 2c3. Figures 3 show LAADF-STEM images and elemental maps of Cr, Fe and Ni by energy dispersive X-ray spectroscopy (EDS), for edge-dislocation (a), partial-dislocation (b) and LCD (c) in 0.19N. STEM-EDS maps were taken from the regions where GPA analysis were carried out in Fig. 2, to correlate elemental distributions and the tensile strained fields. The enrichment of Cr and depletion of both Fe and Ni were confirmed around the tensile strain fields near the dislocation cores, as shown in Figs. 2 and 3. In particular, it is worth noting that Cr enrichments were found corresponding to the triangular tensile strain field in the case of LCD, as shown in Fig. 3c. The compositions of Cr in the matrix, edge-dislocation, partial-dislocation, and LCD were calculated from EDS results to be ~ 25 at%, ~ 35 at%, ~ 35 at%, and ~ 60 at%, respectively, as shown in Supplementary Fig. 1. Abnormal increase in Cr concentration above 60 at% was confirmed in LCD, so that Cr-rich carbon nitride would be precipitated from the LCD. In the case of nitrogen-added austenitic stainless steel, it has been known for the precipitation of Cr_2_N with Shoji-Nishiyama orientation relationship with the surrounding matrix^16,31^. As shown in Fig. 2c1, there was no difference of the crystal structure between the matrix and the Cr-enriched region, so that the abnormal increase in Cr atoms would be caused by the larger and wider tensile strain regions around LCDs.

### Analyses of segregated impurities into dislocations by TEM and APT

Nanostructural characterizations by TEM show that the addition of N stabilized the edge-dislocations, partial-dislocations and LCDs through the assistance of Cr atoms, which led to the localization of dislocations on the slip planes. In this section, correlative TEM/APT analysis was carried out to determine the location of N atoms around the dislocations in 0.19N. In this work, APT cannot unambiguously identify the distribution of N atoms based on the distribution of ions with a mass-to-charge ratio, m/Z, of 14, because ^14^N^1+^ (m/Z = 14) completely overlaps with that of ^28^Si^2+^ (m/Z = 14) in the mass spectrum. However, N is also detected as CrN molecules due to the strong attractive interaction between Cr and N^18,32^. In this study, the presence of the CrN spectra was confirmed in the mass spectrum obtained from 0.19N, as shown in Supplementary Fig. 2, and used for judging the location of N atoms.

**Figure 4:**
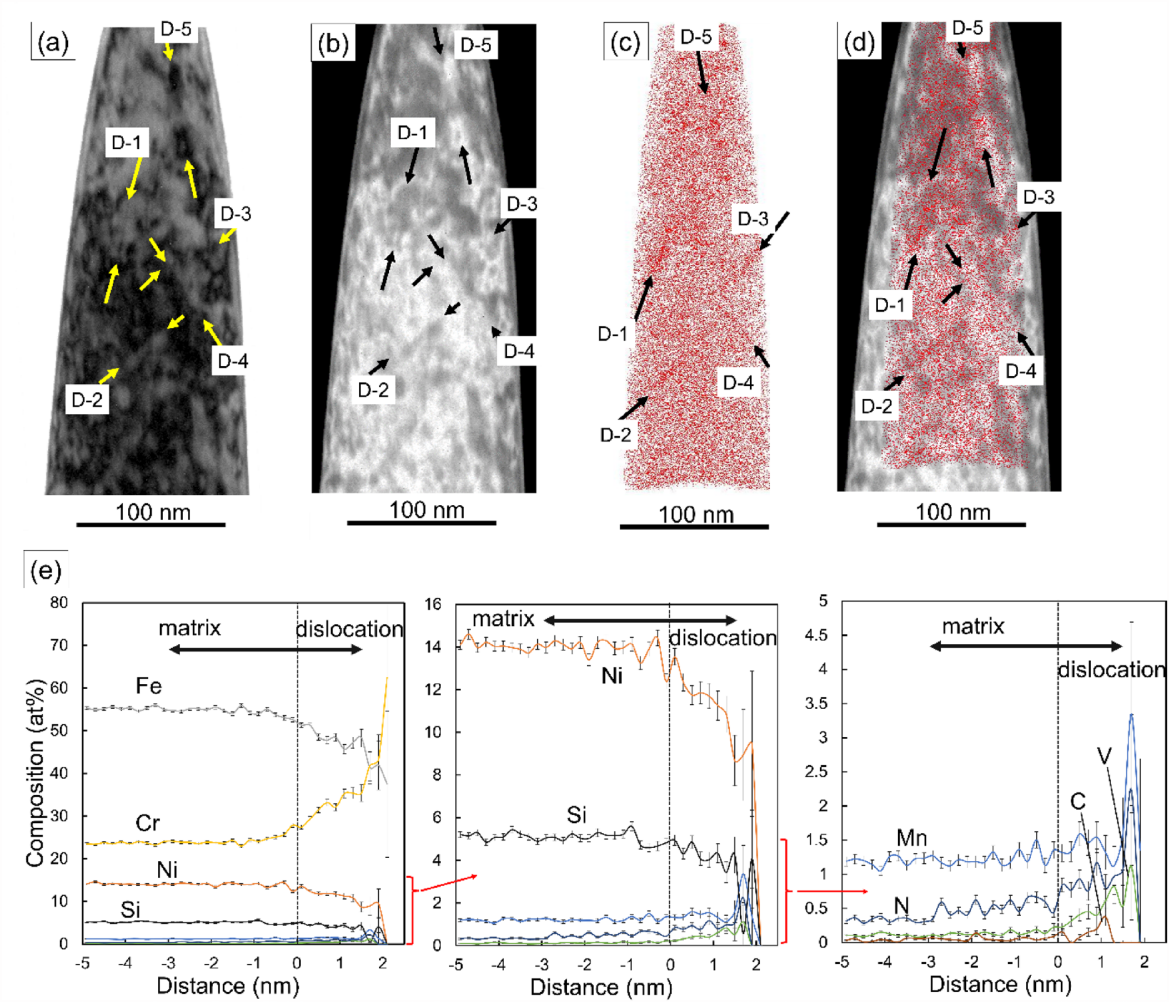
The correlative analysis between TEM and APT of 0.19N. (**a**–**b**) BF-TEM image and its inverted-contrasted image of 0.19N deformed up to 20%. The presences of dislocations are confirmed as indicated by arrows. (**c**) The distribution map of CrN molecules by APT. (**d**) Correlative CrN APT map from the similar region of the sample shown in (**b**). CrN molecules are frequently confirmed around the dislocations. (**e**) Proximity histogram revealing the concentration profile of alloying elements around D-1 in (**a**)–(**d**).

Figure 4a, b show the BF-TEM image and its inverted-contrasted image of 0.19N deformed to 20% strain at 973 K. The linear diffraction contrasts were confirmed as indicated by arrows, corresponding to the dislocations as observed in Fig. 1f. Figure 4c shows the correlative 3D atom map of CrN molecules from the tip of the same sample shown in Fig. 4a, b, d shows the 3D atom map superimposed on the corresponding TEM image, Fig. 4b, showing the CrN enrichment occurs around the dislocations. Figure 4e shows the proximity histogram (proxigram) analyzing the compositional variations from the iso-surface of 27 at% Cr, showing the compositional profile across the D-1. Note that 27 at% Cr was chosen as a threshold for the Cr content to clearly represent Cr-enriched regions. The compositions around dislocations are summarized in Supplementary Table 1, where the enrichment of C, N, V, Cr, and Mn as well as the depletion of Si, Fe, and Ni are seen. Iso-surface concentration maps for N and Cr was shown in Supplementary Fig. 3, showing that the positions of the iso-surfaces were not exactly overlapped between Cr and CrN, as indicated by arrows, so that Cr-rich nitride is not precipitated yet around dislocations, consistent with the TEM results shown in Figs. 2 and 3. Previous studies showed that Cr-rich carbonitrides were precipitated in the dislocation regions^16^, suggesting that the present Cr-enriched regions around the dislocation cores are expected to be the precursors of the carbonitrides.

## Discussion

TEM analyses confirmed that the addition of N changed the dislocation configuration from wavy to planar, where dislocation arrays mainly consisted of edge-dislocation in 0.19N. Atomically-resolved STEM showed that Cr atoms were segregated to the tensile strained field around partial-dislocations and LCDs, as well as edge-dislocations, suggesting that partial-dislocations and LCDs also contribute to the deformation dynamics at 973 K. Ding et al^12^. carried out in-situ straining TEM experiments to characterize the motion of the dislocation arrays in a CrFeCoNiPd high entropy alloy, and showed that the temporary pinning of one of the partial-dislocations resulted in the widely separation of the partials. These results suggest that unstable SFs were temporary stabilized due to the pinning by solid-solution elements, possibly simultaneously stabilizing partial-dislocations and LCD, as well as edge-dislocations at 973 K.

The relationship between N content and SFE has been characterized by TEM, X-ray diffraction (XRD), neutron diffraction (ND) and thermodynamic calculations^21,33,34^, while the effect of N on the SFE is still not obvious. Lee et al. carried out characterizations by TEM, XRD and ND to quantify SFE with different content of N in Fe-15 wt%Mn-2 wt%Cr-0.6 wt%C steels^33^. They showed that N addition increased SFE^33^, consistent with a previous study by Ojima et al^34^. While Kawahara et al. used TEM and showed that N addition decreased SFE in the steels used in this study^21^. As shown in Fig. 2b1–b3 and 3b, Cr enrichment was confirmed in the tensile strained region around the edge of SFs, caused by the elastic interaction between SFs and Cr atoms. Smith et al. performed a detailed STEM-EDS analysis in Ni-based superalloys^29^ and showed that Co and Cr atoms were enriched in both the edge and surface regions of SF, whereas Mo atoms were enriched only in the surface region via the Suzuki effect. In the case of the present alloy, the N addition may have reduced the SFE through the elastic interaction between N atoms and SFs.

APT analysis revealed that N and Cr atoms were segregated to the dislocations, indicating that N and Cr mutually contributed to the dislocation dynamics during high-temperature deformations. N and Cr atoms are present as interstitials and substitutional atoms, respectively, in the FCC matrix and the diffusion rate of the N atom is much higher than that of the Cr atom. Hence, the N segregates to the moving dislocation, resulting in the decrease of the mobility of the dislocations, followed by the segregation of Cr atoms during the high-temperature deformations.

As shown in Figs. 2, 3, and 4, TEM and APT showed that C, N, V, Cr, and Mn atoms were enriched in the vicinity of dislocation cores. C and N atoms were found segregated around the dislocations as interstitials, so they should have contributed to strain relief^25,26^. On the other hand, the enrichment of V, Cr, and Mn are caused by the attractive chemical interactions, and the depletion of Si and Ni would probably be attributed to the repulsive chemical interactions with C and N^18^. These results suggest that I–S effect contributed to the interaction mechanisms as well as the origin of the planar slips in the case of the N-added austenitic stainless steels deformed at 973 K, as proposed by Monma et al^25,26^.

There have been several studies of Cr–N pairs by TEM, APT, mechanical testing and extended X-ray absorption fine structure investigations^32,35,36^, while the presence of Cr–N pairs is still under debate. Previous APT studies of Mo–N containing stainless steels showed that 59% of Mo was detected as MoN molecule, which is around twelve times higher than the fraction of N atoms surrounding a Mo atom as its six nearest neighbors in a random solid solution, suggesting the presence of Mo–N pair^37^. Since Cr content in this study is so high (~ 20 at%), most N atoms should exist as nearest neighbors to Cr atoms. If N atoms occupy octahedral sites, ~ 3.6 Fe, ~ 0.36 Si, ~ 0.007 V, ~ 1.2 Cr and ~ 0.049 Mn atoms should be present around one N atom on average in the matrix, making it difficult to distinguish Cr atoms existing as CrN molecules from those having N atoms as nearest neighbors. On the other hand, in the case of V, the count of ^51^V^14^N^2+^ peak at 32.5 m/Z and ^51^V^2+^ peak at 25.5 m/Z are 110 and 866, respectively, indicating that about 11% of V atoms are detected as VN molecule. In a random solution, 4.38 at% of N atoms occupy the octahedral sites around a V atom, less than half of the fraction of VN molecules, possibly indicating the presences of V–N pairs. As shown in Fig. 4e, proxigram exhibited the enrichment of V and N atoms around the dislocations, where V–N pairs would be formed.

**Figure 5:**
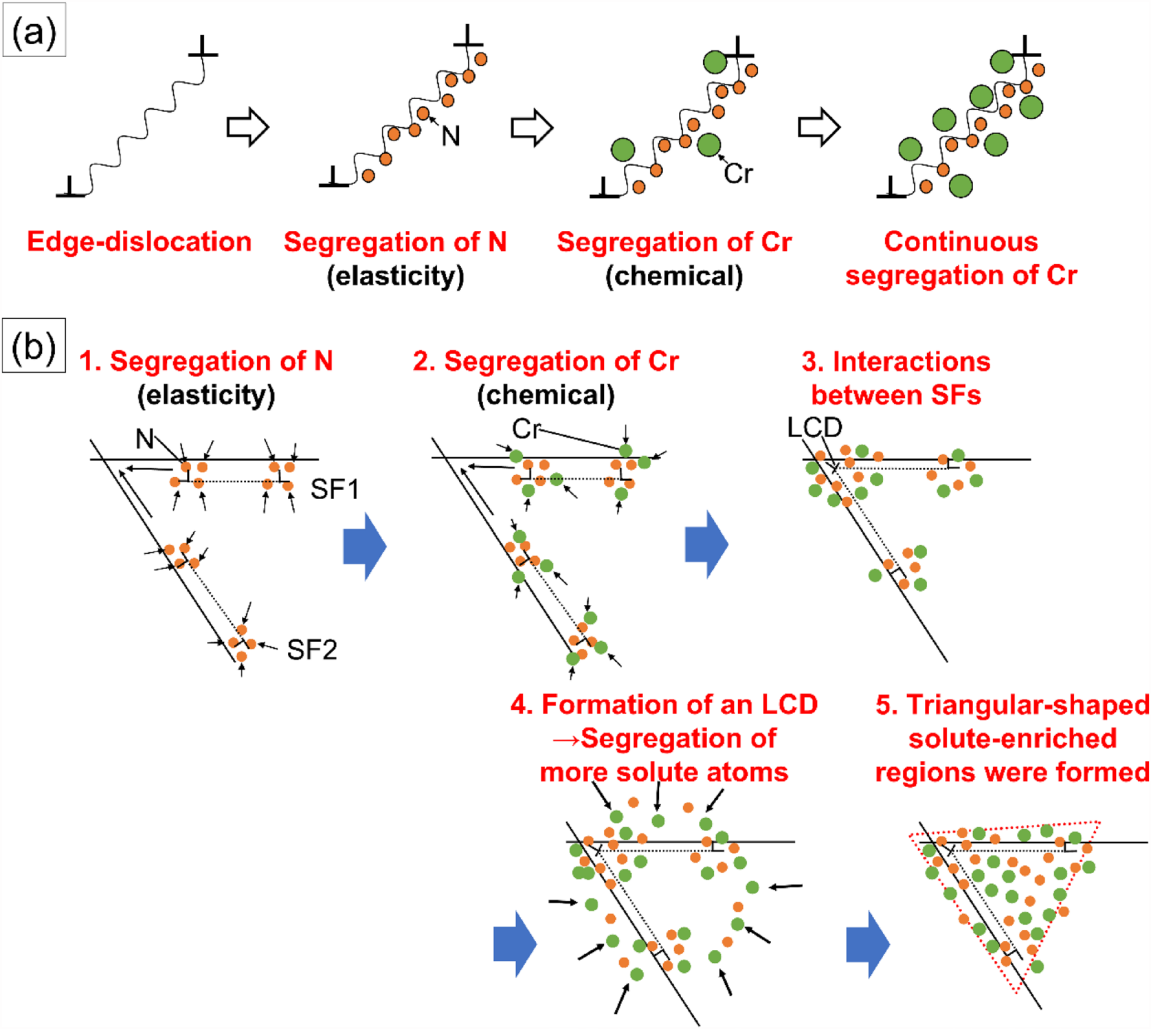
Schematic diagrams for the dynamics of the dislocations in the N-added austenitic stainless steels deformed at 973 K. (**a**) The interaction mechanisms between edge-dislocations and N–Cr pairs. (**b**) The interaction mechanisms between partial-dislocations/LCDs and N–Cr pairs. N–Cr pairs stabilize edge-dislocations, partial-dislocations and LCDs via I–S effect, contributing to the origin of the planar slips.

The interaction mechanisms between edge-dislocations and solute elements are summarized in Fig. 5a. Interstitial N atoms elastically interact with the edge-dislocations at the earlier stage, resulting in the mobility reduction of the dislocations^25,26^. Subsequently, substitutional Cr atoms are gradually segregated to the dislocations due to the chemical interaction with N, namely the I–S effect^18,25,26^. The interaction mechanisms between partial-dislocations/LCDs and solute elements are summarized in Fig. 5b, where N atoms stabilize the partial-dislocations via strain reliefs. Subsequently, Cr atoms were enriched around the dislocations, similarly to the case of the edge-dislocations, and the I–S effect contributes to the dragging resistance for the motion of partial-dislocations. Finally, LCDs are formed via the intersections between partial-dislocations, resulting in the triangular enrichment of N and Cr atoms through the strain relaxation process. Edge-dislocations, partial-dislocations, and LCDs control the whole high-temperature deformation behavior in the case of 0.19N. These dislocations cannot cross-slip, resulting in the localization of dislocations on the slip planes and the excellent work-hardenability.

## Conclusion

In summary, we have carried out the correlation analyses between solute elements and dislocation characteristics in the austenitic stainless steels deformed at 973 K. TEM analysis clarified that the addition of N caused the planar-slips, which consisted of edge-dislocation, partial-dislocation, and LCD. STEM analysis showed that Cr atoms were segregated to the tensile strain field around the dislocation cores, and correlative TEM/APT analysis clarified the segregation of C, N, V, Cr and Mn atoms were enriched around the dislocations. The addition of N stabilizes these dislocation structures through the I–S effect, resulting in superior work-hardenability via the planar-slips.

## Methods

### Materials and tensile test

Si-added austenitic stainless steels, Fe–20Cr–14Ni–0.05C–3Si–0.8Mn–0.1Mo–0.2Cu–0.1V–0.02Al–0.003O–0.03P–0.0007S steels with 0.01, 0.09 and 0.19 of N in wt% or (Fe–20Cr–12Ni–0.21C–6.0Si–0.81Mn–0.05Mo–0.17Cu–0.11V–0.036Al–0.009O–0.055P–0.001S steels with 0.039, 0.35 and 0.73 of N in at%), were fabricated by vacuum melting. These steels were forged at 1523 K, heated at 1423 K for 3.6 ks, heat-rolled, and then annealed at 1423 K for 60 s, followed by air-cooling. These quenched steels were cold-rolled and then annealed at 1423 K for 60 s.

Tensile tests were carried out at 973 K with a strain rate of 1.4 × 10^−3^ s^−1^ on the cold-rolled and annealed steels to evaluate the influence of N addition on the mechanical properties of the steels. The tensile test specimen with gauge dimensions of 35 × 10 × 1 mm (length × width × thickness) was machined, and the loading axis was parallel to the cold-rolling direction.

### TEM sample preparation and image acquisition

TEM and STEM samples were cut from the steels subjected to 5% tensile strain with a strain rate of 5.0 × 10^−5^ s^−1^, and the thin foil specimens were electropolished using the twin-jet method in an electrolyte of 5 mol% of HClO_4_ and 95 mol% of CH_3_COOH. TEM observation was carried out in a JEM-3200FSK (JEOL, Japan) operated at 300 kV, where weak-beam dark-field (DF) imaging was performed to determine the characteristics of dislocations^38^. Moreover, STEM observations were carried out in a JEM-ARM200CF (JEOL, Japan) equipped with a cold field emission gun and a spherical aberration corrector (CEOS, Germany) for the electron probe, operating at 80–120 kV. The convergence semi-angle was 25 mrad and the collection semi-angle was 33 to 131 mrad for low-angle annular-dark field (LAADF)^39^. All atomically-resolved STEM images were acquired by aligning 15 rapidly recorded 1024 × 1024 pixels per frame with 2 μs per pixel using a non-rigid registration method to minimize the specimen drift and improve the image quality^40^.

### STEM-EDS measurements and GPA

Energy dispersive X-ray spectroscopy (EDS) was used to acquire the elemental distribution maps around the dislocations observed in 0.19N using a twin silicon drift detector with a collection solid angle of 2.0 sr. Chemical compositions of Fe, Cr, and Ni segregated around the dislocations were calculated quantitatively from the raw-count EDS maps by the Cliff-Lorimer method.

Geometrical Phase Analysis (GPA) was carried out to evaluate the strain field to study the origin of the contrasts and elemental segregations around the dislocations^41^. GPA was performed based on the aligned atomically-resolved LAADF-STEM images using a GPA plug-in package (HREM Research Inc.), which was implemented in the Digital Micrograph software (Gatan).

### Correlative TEM and APT analysis

A correlative TEM/APT analysis was conducted using an FEI Tecnai 20 TEM and a 5000 XS local electrode atom probe (LEAP). Site-specific lift-out of APT needles from a grain was performed so that the analysis direction for APT and TEM directions were parallel to the < 111 > and < 011 > directions, respectively, using an FEI Helios G4 dual-beam focused-ion-beam (FIB) system. Annular FIB milling was performed using a low acceleration voltage of 16 kV with final polishing at 2 kV to eliminate the damaged layer due to the Ga-ion beam.

TEM analysis was initially carried out under a two-beam condition, followed by APT analysis in voltage mode at a specimen temperature of 30 K. The detection rate, pulse fraction, and pulse rate were 0.5%, 25%, and 500 kHz, respectively. Data reconstruction was carried out using CAMECA IVAS 3.8.8 software.

### Supplementary Information

Supplementary Information.

## Data Availability

The datasets generated during the current study are available from the corresponding authors on request.

## References

[1] Young S-W (2021). Effect of alloying elements on the high-temperature tempering of Fe-0.3N martensite. Acta Mater..

[2] Marioara CD (2007). The effect of Cu on precipitation in Al-Mg-Si alloys. Philos. Mag..

[3] Wang Z (2020). Formation mechanism of κ-carbides and deformation behavior in Si-alloyed FeMnAlC lightweight steels. Acta Mater..

[4] Jiang S (2017). Ultrastrong steel via minimal lattice misfit and high-density nanoprecipitation. Nature.

[5] Masumura T, Tsuchiyama T (2021). Effect of carbon and nitrogen on work-hardening behavior in metastable austenitic stainless steel. ISIJ Int..

[6] Matsuda K (2019). Effect of copper addition on precipitation behavior near grain boundary in Al-Zn-Mg Alloy. Mater. Trans..

[7] Lervik A, Wenner S, Lunder O, Marioara CD, Holmestad R (2020). Grain boundary structures and their correlation with intergranular corrosion in an extruded Al-Mg-Si-Cu alloy. Mater. Charact..

[8] Jin WZ, Kokawa H, Wang ZJ, Sato YS, Hara N (2010). Improvement of transpassive intergranular corrosion resistance of 304 austenitic stainless steel by thermomechanical processing for twin-induced grain boundary engineering. ISIJ Int..

[9] Toro A, Misiolek WZ, Tschiptschin AP (2003). Correlations between microstructure and surface properties in a high nitrogen martensitic stainless steel. Acta Mater..

[10] Miyamoto G, Tateyama Y, Uesugi T, Hayasaka Y, Furuhara T (2021). Solute cluster-induced precipitation and resultant surface hardening during nitriding of Fe-Al-V alloys. Scripta Materialia.

[11] Zhang R (2020). Short-range order and its impact on the CrCoNi medium-entropy alloy. Nature.

[12] Ding Q (2019). Tuning element distribution, structure, and properties by composition in high-entropy alloys. Nature.

[13] Findlay SD, Azuma S, Shibata N, Okunishi E, Ikuhara Y (2011). Direct oxygen imaging within a ceramic interface, with some observations upon the dark contrast at the grain boundary. Ultramicroscopy.

[14] Bian MZ (2018). Bake-hardenable Mg-Al-Zn-Mn-Ca sheet alloy processed by twin-roll casting. Acta Mater..

[15] Wang H, Zhang X, Yan D, Somsen C, Eggeler G (2018). Interface dominated cooperative nanoprecipitation in interstitial alloys. Nat. Commun..

[16] Lo KH, Shek CH, Lai JKL (2009). Recent developments in stainless steels. Mater. Sci. Eng. R Rep..

[17] Simmons JW (1996). Overview: High-nitrogen alloying of stainless steels. Mater. Sci. Eng. A.

[18] Gavriljuk VG, Berns H (2013). High Nitrogen Steels: Structure, Properties, Manufacture, Applications.

[19] Müllner P, Solenthaler C, Uggowitzer P, Speidel MO (1993). On the effect of nitrogen on the dislocation structure of austenitic stainless steel. Mater. Sci. Eng. A.

[20] Pekin TC, Gammer C, Ciston J, Ophus C, Minor AM (2018). In situ nanobeam electron diffraction strain mapping of planar slip in stainless steel. Scripta Mater..

[21] Kawahara Y, Teranishi R, Takushima C, Hamada J, Kaneko K (2021). Effect of nitrogen addition on the stacking-fault energies in Si-added austenitic stainless steel. ISIJ Int..

[22] Gerold V, Karnthaler HP (1989). On the origin of planar slip in f.c.c. alloys. Acta Metall..

[23] Kato M, Katsuka S, Okamine S, Sato A (1986). Deformation behaviour and microstructure of Cu-10Ni-6Sn spinodal alloy single crystals. Mater. Sci. Eng..

[24] Sato A, Tamura K, Ito M, Kato M, Mori T (1993). In situ observation of moving dislocations in a Cu-10Ni-6Sn spinodal alloy. Acta Metall. Mater..

[25] Monma K, Sudo H, Ogita H (1965). On the chemical interaction between interstitial atoms and substitutional atoms affecting the strength of iron-chromium and iron-molybdenum alloys. J. Jpn. Inst. Metals Mater..

[26] Terada D, Yoshida F, Nakashima H, Abe H, Kadoya Y (2002). In-situ observation of dislocation motion and its mobility in Fe-Mo and Fe-W solid solutions at high temperatures. ISIJ Int..

[27] Saenarjhan N, Kang J-H, Kim S-J (2019). Effects of carbon and nitrogen on austenite stability and tensile deformation behavior of 15Cr-15Mn-4Ni based austenitic stainless steels. Mater. Sci. Eng. A.

[28] Hirth JP, Lothe J (1992). Theory of Dislocations.

[29] Smith TM (2018). Segregation and phase transformations along superlattice intrinsic stacking faults in Ni-based superalloys. Metall Mater. Trans. A.

[30] Xu XD (2018). Transmission electron microscopy characterization of dislocation structure in a face-centered cubic high-entropy alloy Al0.1CoCrFeNi. Acta Mater..

[31] Ochi M (2018). Nanostructural analyses of intra- and intergranular precipitates in high-temperature heat-treated nitrogen-added austenitic stainless steel. ISIJ Int..

[32] Martinavičius A (2015). Atom probe tomography characterization of nitrogen induced decomposition in low temperature plasma nitrided 304L austenitic stainless steel. Mater. Lett..

[33] Lee S-J (2014). The effect of nitrogen on the stacking fault energy in Fe–15Mn–2Cr–0.6C–xN twinning-induced plasticity steels. Scripta Mater..

[34] Ojima M (2009). Weak beam TEM study on stacking fault energy of high nitrogen steels. Steel Res. Int..

[35] Xie Y, Miyamoto G, Furuhara T (2023). High-throughput investigation of Cr-N cluster formation in Fe-35Ni-Cr system during low-temperature nitriding. Acta Mater..

[36] Oddershede J, Christiansen TL, Ståhl K, Somers MAJ (2010). Extended X-ray absorption fine structure investigation of nitrogen stabilized expanded austenite. Scripta Mater..

[37] Wahlberg G, Rolander U, Andrén H-O (1989). Interaction between nitrogen and substitutional elements in the austenitic phase of duplex austenitic-ferritic stainless steels. Proc. High Nitrogen Steels HNS.

[38] Cockayne DJH, Ray ILF, Whelan MJ (1969). Investigations of dislocation strain fields using weak beams. Philos. Mag. J. Theor. Exp. Appl. Phys..

[39] Phillips PJ (2012). Atomic-resolution defect contrast in low angle annular dark-field STEM. Ultramicroscopy.

[40] Jones L (2015). Smart align-a new tool for robust non-rigid registration of scanning microscope data. Adv. Struct. Chem. Imaging.

[41] Hÿtch MJ, Snoeck E, Kilaas R (1998). Quantitative measurement of displacement and strain fields from HREM micrographs. Ultramicroscopy.

