# Supplementary material for: Combined effect of interstitial-substitutional elements on dislocation dynamics in nitrogen-added austenitic stainless steels
Source: Sci Rep. 2024 Feb 22;14:4360. doi: 10.1038/s41598-024-54852-w (PMC10883984; doi:10.1038/s41598-024-54852-w)
Supplement: Supplementary file 1 — Supplementary Information. [file 41598_2024_54852_MOESM1_ESM.docx]

**Supplementary information**

Supplementary Figure 1


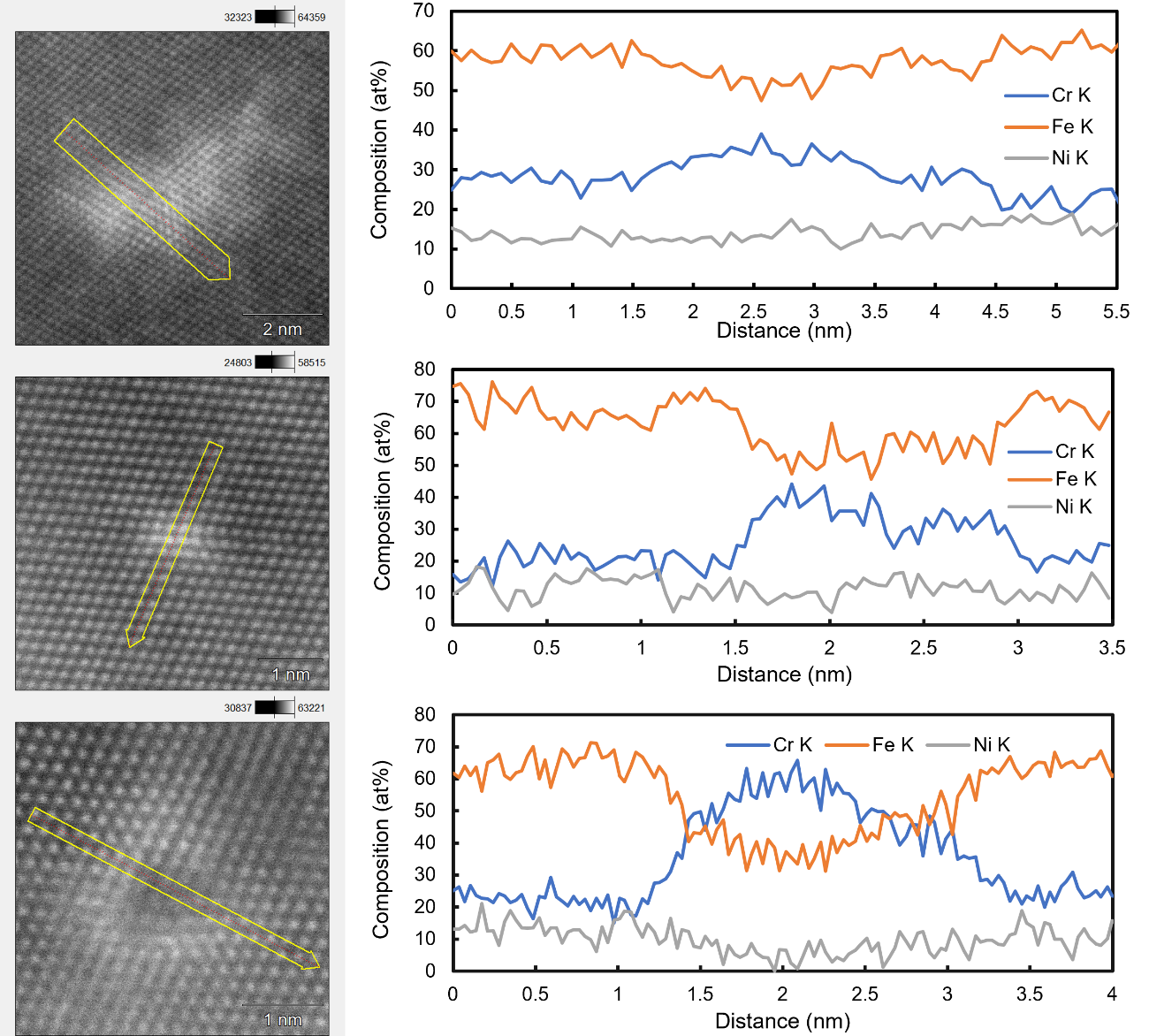


Sup.Fig.1 Compositional analysis of the edge-dislocation, partial-dislocation and LCD in 0.19N. Cr content was measured to be ~25 at%, ~35 at%, ~35 at% and ~60 at% in the matrix, edge-dislocation, SF and LCD, respectively.

Supplementary Figure 2


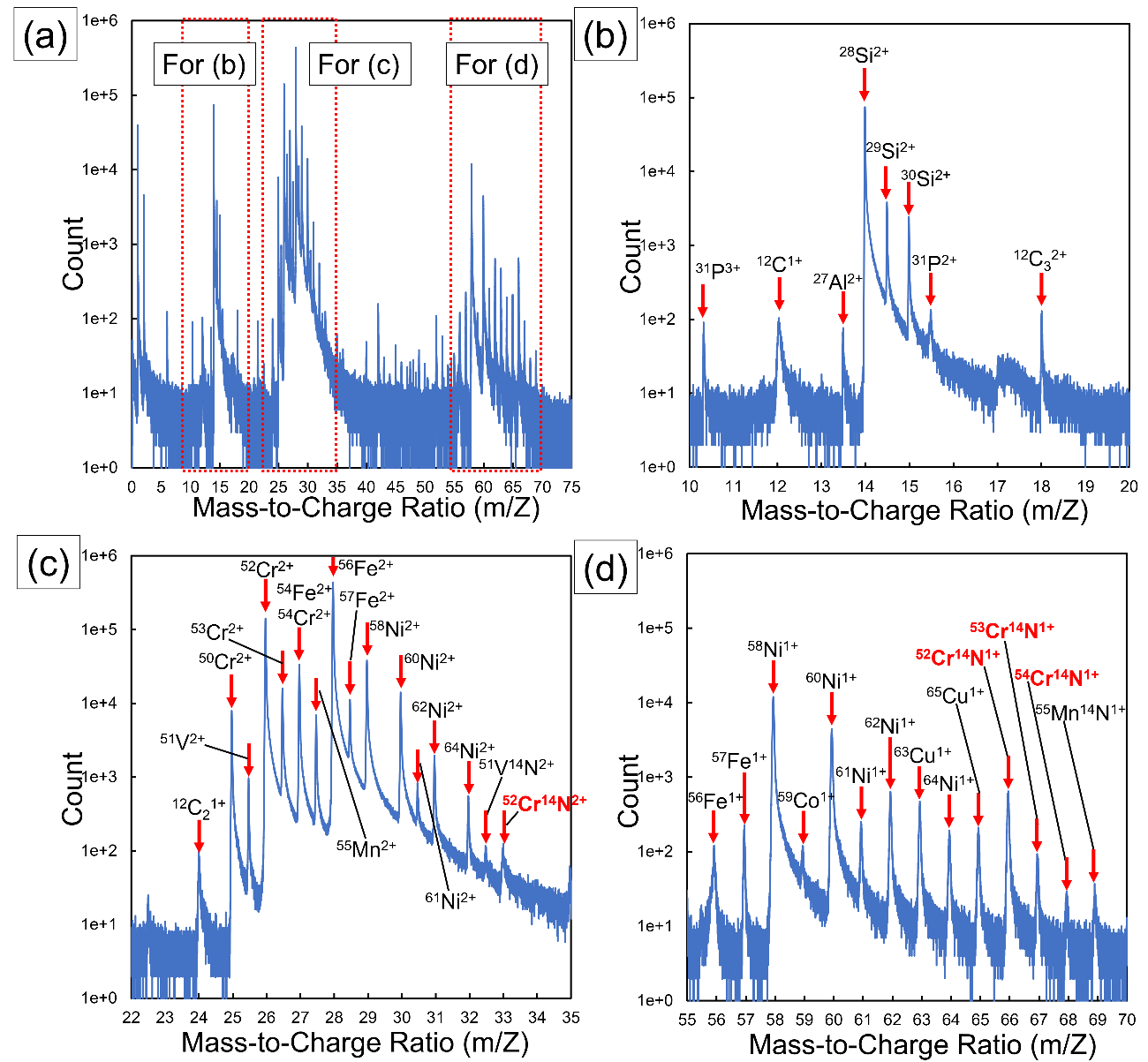


Sup.Fig.2 Mass spectrum obtained from 0.19N deformed up to 20% strain, where N atoms are detected as CrN molecules due to their attractive chemical interactions with Cr atoms.

Supplementary Table 1

Sup.Table 1 Compositions of segregated impurities around the defect by APT (at%).

| **No.** | **Mn** | **Ni** | **Fe** | **Cr** | **Si** | **V** | **N** | **C** |
| --- | --- | --- | --- | --- | --- | --- | --- | --- |
| 1 | 1.12  -3.35 | 8.67  -13.5 | 41.3  -51.3 | 27.4  -41.9 | 0.559  -4.94 | 0.230  -1.12 | 0.758  -2.23 | 0.0777  -0.201 |
| 2 | 0.224  -1.60 | 6.12  -12.2 | 40.8  -52.5 | 26.9  -44.9 | 1.79  -5.69 | 0.218  -0.728 | 0.604  -2.98 | 0.0330  -0.881 |
| 3 | 0.777  -3.79 | 8.55  -13.1 | 41.7  -51.5 | 28.5  -40.2 | 0.758  -4.99 | 0.247  -0.838 | 0.614  -1.30 | 0.0617  -0.240 |
| 4 | 0.667  -1.55 | 7.33  -13.1 | 40.8  -51.3 | 28.2  -42.7 | 1.33  -5.17 | 0.178  -.267 | 0.215  -1.44 | 0.0669  -1.33 |
| 5 | 0.962  -1.42 | 8.40  12.7 | 40.4  -51.2 | 28.6  -42.4 | 3.92  -6.40 | 0.227  -1.26 | 0.646  -1.18 | 0.0568  -0.444 |
| 6 | 0.850  -1.72 | 12.2  -14.7 | 44.7  -53.0 | 27.1  -32.4 | 4.08  -5.77 | 0.262  -0.668 | 0.459  -1.63 | 0.0656  -0.227 |
| Matrix  (-4.9 nm~  -1.5 nm) | 1.20  ±0.06 | 14.1  ±0.25 | 55.1  ±0.43 | 23.7  ±0.26 | 5.12  ±0.15 | 0.114  ±0.02 | 0.394  ±0.10 | 0.0466  ±0.03 |

Supplementary Figure 3


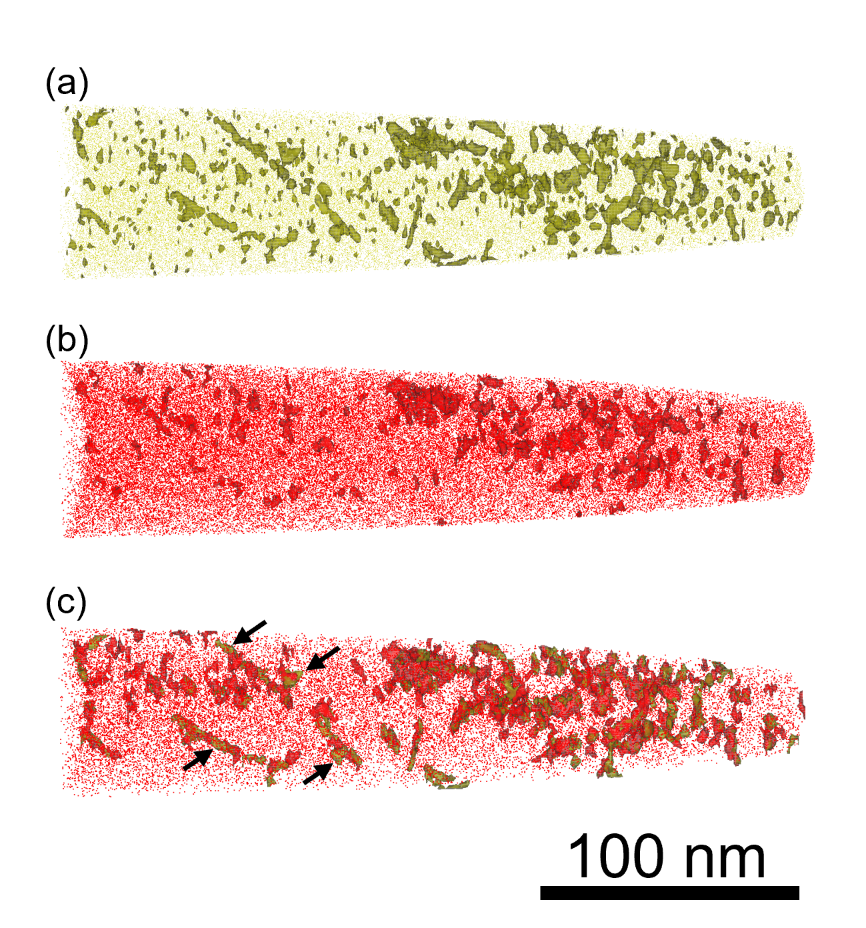


Sup.Fig.3 Iso-surface concentration maps of (a)Cr, (b)CrN and (c)Cr+CrN, where iso-concentrations of Cr and CrN are 20 at% and 0.45 ionic%, respectively.
